# Drug-independent control strategy of clostridial infection in broiler chickens using anti-toxin environmentally friendly multienzymes

**DOI:** 10.1038/s41598-023-32685-3

**Published:** 2023-04-06

**Authors:** Ahmed A. Saleh, Abdelhaleem Hafez, Khairy Amber, AbdelRahman Y. Abdelhady, Heba M. Salem, M. Fathy, M. A. Kamal, Mahmoud Alagawany, Mohammed H. Alzawqari

**Affiliations:** 1 Department of Poultry Production, Faculty of Agriculture, Kafrelsheikh University, Kafrelsheikh, 333516 Egypt; 2 Poultry Production Department, Faculty of Agriculture, Ain Shams University, Hadayek Shoubra, Cairo, 11241 Egypt; 3 Department of Poultry Diseases, Faculty of Veterinary Medicine, Cairo University, Giza, 12211 Egypt; 4 Department of Animal and Poultry Health, Desert Research Centre, Cairo, 11753 Egypt; 5 Department of Veterinary Hygiene and Management, Faculty of Veterinary Medicine, Cairo University, Giza, 12211 Egypt; 6 Department of Poultry, Faculty of Agriculture, Zagazig University, Zagazig, 44511 Egypt; 7 Department of Animal Production, Faculty of Agriculture and Food Sciences, Ibb University, 70270 Ibb, Yemen

**Keywords:** Immunology, Zoology

## Abstract

The study investigated the effect of enzymes as a toxin detoxifier (DETOXIZYME) dietary supplementation on performance during growth, blood chemistry, and immunity under clostridia infection in chickens. A total of 480, day-old male chicks were randomly distributed to four groups, with six replicates of 20 birds each. The first control negative treatment (A) fed the basal formula as commercial feed prepared following the strain's needs, the second control positive group (B) fed the basal formula challenged with *Clostridium perfringens* (*C. perfringens*) type A, the third group (C) fed the basal formula with 100 g DETOXIZYME/ton of feed and challenged with clostridia, and the fourth group (D) fed the control basal formula with 100 g DETOXIZYME/ton of feed. DETOXIZYME dietary supplementation significantly boosted body weight (BW), body weight gain (BWG), feed intake (FI), and European production efficiency factor (EPEF) and improved the feed conversion rate (FCR) of the broilers. The dietary supplementation of DETOXIZYME significantly increased carcass trait and spleen. However, liver and abdominal fat weight significantly decreased compared with clostridia-challenged groups. The values of alanine aminotransferase (ALT), aspartate aminotransferase (AST), uric acid, creatinine, and Malondialdehyde (MDA) were decreased. While calcium, phosphate, zinc, and glutathione peroxidase (GPx) levels were improved in birds that took basal formulas fortified with DETOXIZYME contrary to the other treatment groups during 35 days of age. Plasma total cholesterol, triglyceride, and low-density lipoprotein (LDL) values were reduced versus the other treatment groups. Dietary supplementation of DETOXIZYME increased total protein, albumin, globulin, and Newcastle Disease (ND) immunity titer levels in the overall period compared to other groups. Dietary DETOXIZYME supplementation decreased clostridia and *E. coli* bacteria counts and improved gut morphometry. In conclusion, dietary supplementation of DETOXIZYME had a positive impact on performance, blood biochemistry, immunity, and bacterial counts and improved the gut morphology in broilers under clostridia infection.

## Introduction

To serve as a barrier against viruses, toxins, and other detrimental biochemical effects and to allow for the assimilation of nutrients and liquids, the gastrointestinal tract (GIT) of broilers must be in good health^1,2^. Furthermore, it enhances mucosal immune response^3,4^. Therefore, despite major advancements in feed science and feed processing technologies, researchers and the industry are still looking into new strategies to support feed cleanliness^5–7^.

The recognized endo- and exotoxins that *Clostridium perfringens* bacteria produce enable the clostridia to enter the sensitive intestinal tissues of broilers^8–10^. The tight junction is destroyed, and the barrier function is disrupted when enterotoxins from *C. perfringens* bind to a protein called claudin^11,12^ and adversely impact birds' weight gain, FCR, and crude protein digestibility^13^.

On the other hand, mycotoxin species found in chicken feedstuffs such as aflatoxin or fumonisin impair GIT functions at dosages between 10 and 20 mg/kg^14^. It should be emphasized that under ideal climatic conditions, in the course of storage, mycotoxins can develop both before harvest and after harvest^15^. Liver damage, poor performance, immunological suppression, biochemical, hematological, reproductive, and pathological alterations, as well as mortality, have all been related to broiler intake of aflatoxin^16,17^.

A persistent problem with feed safety is endotoxin and mycotoxin contamination, which causes hepatic impairment, and affects animal productivity, and feed security^18–20^. There are two major approaches for stopping the development of mycotoxins and detoxification: physical methods or chemical procedures, both of which have been widely employed to get rid of mycotoxins^21^. To selectively combine the mycotoxins during the digestion activity and render them non-toxic to the birds, mycotoxicosis could be prevented by adding non-nutritional and natural adsorbent raw material to the diet^22–24^.

Chemical methods used in mycotoxin management have many drawbacks like chemical pollution of feed materials and nutrient deficiency, before being both timewasting and expensive. Enzyme detoxification has the advantage of having high safety with effective and low-cost mycotoxin control^25–27^. Although they only function as bio-converting agents for mycotoxins, bacteria-produced enzymes can operate as mycotoxin detoxifiers^28,29^. Additionally, before mycotoxins are absorbed, bacterial enzymes can transform them into non-toxic metabolites that the animal can consume without causing toxicity^30^. Other enzymes like proteases, carboxypeptidases, and lactonohydralases can detoxify mycotoxins into non-toxic byproducts^29^. The effectiveness of mycotoxin breakdown processes was established by applying microbial enzymes and metabolites^31,32^. Broilers' growth performance, immunological response, ileal counts of *Clostridium perfringens*, intestinal lesions, and serum alpha-toxin antibodies all improved by adding dietary anti-toxin multienzymes^33^. Moreover, using feed multienzymes to reduce *C. perfringens* in broiler feed is environmentally friendly^34^.

Likewise, Jia et al. confirmed that the impacts of the *C. perfringens* challenge were lessened by adding multienzymes to broiler diets, which enhanced growth performance^35^. Furthermore, it is asserted that using enzyme compounds comprising carbohydrases and proteases will enhance the broiler chickens' use of calcium, phosphorus, protein, and energy^36,37^.

A gram-positive, anaerobic bacterium enzyme called Eubacterium BBSH797's an epoxidase can enzymatically change deoxynivalenol into the harmless metabolite deepoxydeoxynivalenol DOM-1^29,30^. Based on Ademola et al., broilers fed a ration fortified with DETOXIZYME demonstrated synergetic results of detoxification enzymes (such as esterase, peptidase, epoxide reductase, and carbonyl aflatoxin B1 reductase) and improved final BW and BWG^38^. As a result, multienzymes are recommended for use as detoxifiers and growth promoters in chickens. Our hypothesis is the supplementation of specific microbial enzymes in feed, could be used as a preventive method against poultry clostridial infection either directly through the destruction of clostridial toxins or indirectly through the improvement of gut health, blood chemistry, and destruction of mycotoxins. Therefore, this study was performed to estimate the impact of toxins detoxifier multienzymes supplement on performance, blood chemistry, and immunity under experimental clostridia challenge in broilers.

## Material and methods

### Ethical statement

The experiment was accepted by the Ethics Committee of the Local Experimental Animals Care Committee and performed under the guidelines of the Department of Poultry Production, Faculty of Agriculture, Kafrelsheikh University, Egypt and all methods were performed in accordance with the relevant guidelines and regulations (Approval number: 4/2016 EC). The study was conducted following ARRIVE guidelines.

### Birds and experimental design

Overall, 480-day-old male chickens (Cobb 500) were kept in bins (10 birds per m^2^) and allocated into 4 experimental groups randomly with 6 replicates (20 birds to each) to match the average live body weight in each treatment. The trial treatments comprise rations formulated based on the feed requirements of Cobb^39^ for male chickens, with a 3-phase feeding strategy (starter formula, 0–10 days; grower formula, 11–24 days; and finisher formula, 25–35 days). The initial control negative treatment (A) took the basal formula as a commercial ration composed according to the breed needs, the other control positive treatment (B) took the basal diet challenged with clostridia, the third experimental treatment (C) took the basal formula with 100 g a blend of specific natural detoxifying enzymes (DETOXIZYME, CEVA Polchem, Pvt. Ltd. India and it was obtained from 3A Pharma, Tanta, Egypt, Reg. No. 6121, DETOXIZYME content protease activity from *Bacillus licheniformis* 2000 IU/g)/ton of feed and challenged with clostridia, while the fourth group (D) took the basal formula with 100 g DETOXIZYME/ton of feed. The experimental diet's ingredients were chosen to be following the demands of the Cobb 500 broiler chicken strain^39^, as shown in Table 1. The initial diets were in the shape of crumbles, and the birds could eat them whenever they wanted. Diets for growers and finishers, however, came in pellet form. A house with open windows and a cycle of 23 h of light and 1 h of darkness was used to maintain the route. Daily indoor humidity and temperature were maintained at 60 to 70% and 24 to 26 °C, respectively. Experimental diets were made available from one day to 35 days of age.

**Table 1:** Composition of the experimental starter, grower, and finisher diets.

Ingredient, g/kg	Starter	Grower	Finisher
	(1–10 days)	(11–25 days)	(26–35 days)
Yellow corn	507	548	578
Soybean meal, 46%	370	317	280
Corn gluten meal, 60%	38	50	50
Soya oil	17	21	31
Calcium carbonate	14.0	13.8	12.6
Dicalcium phosphate	20.0	17.5	16.0
Salt	2.3	2.4	2.3
Sodium sulfate	1.8	1.6	1.6
Dl Methionine, 99%	2.7	2.0	1.9
l-Lysine HCl, 98%	2.5	2.3	2.2
l-Threonine	1.1	0.7	0.6
Choline chloride, 60%	0.8	0.8	0.8
Premix*	2	2	2
Anticoccidia	0.2	0.2	0.2
Anticlostridia	0.1	0.1	0.1
Antimycotoxin biology	0.25	0.25	0.25
Silica	1	1	1
Chemical analysis on DM basis			
AME kcal	3000	3040	3140
Crude protein, %	23.0	21	19
Fat, %	6.3	4.5	6.9
Digestible LYS, %	1.28	1.24	1.15
Digestible M and C, %	0.95	0.92	0.87
Digestible THR, %	0.86	0.83	0.77
Digestible ARG, %	1.37	1.33	1.25
Digestible ILE, %	0.90	0.87	0.85
Digestible LEU, %	1.87	1.83	1.84
Digestible VAL, %	0.96	0.93	0.91
Calcium, %	0.96	0.96	0.87
Available P, %	0.48	0.48	0.44
Sodium, %	0.16	0.16	0.16
Chloride, %	0.23	0.23	0.23

*Hero mix® (Hero pharm, Cairo, Egypt). Composition (per 3 kg): Vitamin A 12,000,000 IU, vitamin D3 2,500,000 IU, vitamin E 10,000 mg, vitamin K3 2000 mg, vitamin B1 1000 mg, vitamin B2 5000 mg, vitamin B6 1500 mg, vitamin B12 10 mg, niacin 30,000 mg, biotin 50 mg, folic acid 1000 mg, pantothenic acid 10,000 mg, manganese 60,000 mg, zinc 50,000 mg, iron 30,000 mg, copper 4000 mg, iodine 300 mg, selenium 100 mg, and cobalt 100 mg. Diet ingredients and final feed diets were analyzed by chemical analysis in the Adisseo company lab, Antony, France.

### Challenge bacteria

The birds in both groups B and C were challenged at 14 days old for two successive days with pathogenic *C. perfringens* type A identified strain, which was obtained from the Department of Poultry Diseases, Faculty of Veterinary Medicine, Cairo University. Each bird was challenged via crop gavage with 1 ml cooked meat broth containing 4 × 10^8^ colonies forming unit (CFU) freshly prepared *C. perfringens* type A (18 h anaerobic incubation period at 37℃) as described by Salem et al.^40^.

### Birds' performance and organs’ weights

Each week, the weight of each bird was recorded. Nevertheless, during the trial period, feed consumption was assessed daily (collectively per pen). On day 32, every bird was weighed individually and arranged from lightest to heaviest. To conduct the digestibility experiment, 12 male birds, all of the same weight, were relocated to separate cages. The weights of the carcass, muscle of the breast, muscle of the thigh, the liver, the gizzard, the heart, the spleen, and fat of the abdomen were then measured after the birds had been slaughtered and dissected. A ratio of the weight of the body was used to weigh and describe each organ. Just before slaughter, blood samples were drawn from the vein of the wing, gathered in heparinized test tubes, and the plasma was separated immediately by centrifugation (3000 rpm for 20 min at 5 °C). The plasma was kept at − 20 °C for additional assessment.

### Crude protein digestibility

For the crude protein digestibility testing, during the final three days of the experiment, droppings from each cage replicate were collected and weighed. Over these three days, each day, the birds' feed consumption and weight were measured, and the excrement they passed was gathered, weighed, and put in a freezer. All samples were dried for 24 h at 60 °C in a drying oven after the digestibility test. Following homogenization, the fully dried samples were finely powdered for testing following Lim et al.^41^. The Kjeldahl process was applied to determine the crude protein substance in the diet and excreta and the nitrogen's digestibility (CP, Method 968.06).

### Blood parameters’ analysis

Blood samples for alanine aminotransferase (ALT), aspartate aminotransferase (AST), Malondialdehyde (MDA), and glutathione peroxidase (GPx) concentrations were measured using a commercially available colorimetric kit (ALT, AST, MDA, and GPx; Egyptian Company for Biotechnology). A spectrophotometer (Unico UV—2000; Spectra Lab Scientific Inc., USA) calibrated at 545 nm wavelength was used to measure the absorbance (Saleh et al. 2019). According to the instructions specified by the producer, the levels of uric acid and creatinine were established colorimetrically using commercially available kits (Diamond Diagnostics, Egypt) Saleh^42^. Blood contents including calcium, phosphate, and zinc were analyzed and determined using gas–liquid chromatography (GLC) following Lim et al.^41^.

### Plasma total lipids analysis

Using commercially available kits from Diamond Diagnostics in Egypt, blood samples of total cholesterol, triglycerides (TG), low-density lipoprotein (LDL) cholesterol, and high-density lipoprotein (HDL) cholesterol were tested calorimetrically per the manufacturer's instructions^42^.

### Immunity evaluation

Using commercially available kits (Diamond Diagnostics, Egypt), total protein, albumin, and globulin, were quantified calorimetrically by the instructions provided by the manufacturer^42^. The hemagglutination inhibition test was applied to measure the serum antibody titer for Newcastle disease (ND) using conventional techniques that were approved by Steer^43^.

### Bacteriological counting

Plate count agar (Merck, 1.05463, Darmstadt, Germany) was used to count the total bacterial count (TBC) for 2 days at 35 °C. *E. coli* and *Clostridium Perfringens* colonies count: 1 g from each sample was diluted 1 to 9 times (wt/vol) in sterile PBS before being serially diluted 10 times. With a slight adjustment, the colony counting was carried out following Quinn^44^. In Brief, the samples were diluted and then incubated anaerobically for one day at 37 °C in Reinforced Clostridia Agar Medium (Oxoid) for *C. perfringens* also, dilutions were inoculated on EMB medium for *E. coli* colonies count and incubated aerobically at 37 °C for one day.

### Histopathological examination

Five birds from each group were randomly chosen, and the abdomen was dissected, to obtain tissue samples from the duodenum. Samples of the liver were placed in a 10% formaldehyde solution for 24 h whereas intestinal samples were placed in Bouin's solution for eighteen hours. Following fixation, samples of the tissue were dehydrated in ethyl alcohol at increasing concentrations (from 70 percent to absolute alcohol), cleaned in xylene, and got ready for histological analysis. Hematoxylin and eosin were used to stain sections of 4–5 µm thickness for histological analysis according to Bancroft et al.^45^.

### Data analysis

The acquired data were analyzed utilizing SPSS statistical software version 26 (IBM SPSS stats for Windows Armonk, NY: IBM Corp). Using Tukey's multiple comparison test based on (P < 0.05) the significance of all mean differences was examined.

## Results

### Birds’ performance parameters

Table 2 illustrates the effects of dietary supplementation of DETOXIZYME treatments on final BW (FBW), BWG, FI, FCR, death rate, EPEF, and crude protein (CP) digestibility in Cobb 500 broilers under clostridia infection during the experimental period. Broilers infected with *Clostridia perfringens* showed bad performance signs including significant decreases in FBW, BWG, FI, FCR, EPEF, and a higher death rate. Feed DETOXIZYME supplementation significantly increased FBW, BWG, FI, and EPEF, and improved the FCR rate of the broilers during experimental periods of age. While dietary DETOXIZYME treatments significantly reduced (P < 0.05) the mortality rate as opposed to B and C treatment groups during 35 days of age.

**Table 2:** Effect of DETOXIZYME supplementation on growth performance under clostridia infection in Cobb 500 broilers.

Item	Experimental diets				*P*-value
	A	B	C	D	
Initial body weight, g	42.3 ± 0.15	42.7 ± 0.14	42.7 ± 0.23	42.6 ± 0.14	0.2200
Body weight, 35d, g	2281 ± 18^ab^	2062 ± 28^c^	2221 ± 20^b^	2308 ± 22^a^	0.0001
Body weight gain, 35d, g	2239 ± 18^ab^	2019 ± 28^c^	2179 ± 20^b^	2266 ± 21^a^	0.0001
Feed intake, 35d, g	3532 ± 43^a^	3349 ± 16^b^	3489 ± 22^a^	3515 ± 25^a^	0.0007
FCR, 35d	1.549 ± 0.02^b^	1.626 ± 0.02^a^	1.571 ± 0.02^ab^	1.523 ± 0.01^b^	0.0170
Mortality, 35d, %	1.66 ± 1.05^c^	4.166 ± 0.83^a^	3.33 ± 1.05^b^	2.5 ± 1.11^c^	0.0390
EPEF, index	406.2 ± 21^a^	339.9 ± 18^b^	383.1 ± 21^ab^	414.5 ± 17^a^	0.0410
CP digestibility, %	74.1 ± 2.6^a^	61 ± 5.6^b^	71.4 ± 3.4^ab^	74.7 ± 1.4^a^	0.0270

^a–c^The means values placed at the rows by different superscript letters are significantly different (P < 0.05). Values are expressed as means ± standard error. Abbreviations: (A) control negative (basal diet), (B) control positive (basal diet supplemented with clostridia infection), (C) control positive group diet with 100 g DETOXIZYME/ton of feed, (D) control negative diet group with 100 g DETOXIZYME/ton of feed, (EPEF) European production efficiency factor.

### Carcass and internal organs weight

Table 3 demonstrates the influence of the dietary supplementation DETOXIZYME on carcasses and weight of the internal organs in Cobb 500 chickens under clostridia challenge during the trial period. The supplementation of DETOXIZYME did not alter the breast, thigh, gizzard, and heart. However, carcass and spleen were significantly improved in the DETOXIZYME treated group as opposed to clostridia infection groups (P < 0.05). Although, birds consumed 100 g/ton of DETOXIZYME had the lowest liver and abdominal fat weight when contrary to clostridia infection treatment groups.

**Table 3:** Effect of DETOXIZYME supplementation on carcass and internal organs weight under clostridia infection in Cobb 500 broilers.

Item	Experimental diets				*P*-value
	A	B	C	D	
Carcass, g/100 g BW	65.6 ± 0.7^a^	63.3 ± 0.6^b^	65.5 ± 0.5^a^	65.7 ± 0.7^a^	0.046
Breast muscle, g/100 g BW	23.2 ± 0.6	21.9 ± 0.4	22.5 ± 0.8	23.2 ± 0.7	0.422
Thigh muscle, g/100 g BW	16.0 ± 0.7	15.3 ± 0.3	15.5 ± 0.3	16.8 ± 0.7	0.290
Gizzard, g/100 g BW	0.86 ± 0.05	0.85 ± 0.02	0.88 ± 0.03	0.87 ± 0.08	0.291
Liver, g/100 g BW	1.549 ± 0.02^b^	1.626 ± 0.02^a^	1.571 ± 0.02^ab^	1.523 ± 0.01^b^	0.017
Spleen, g/100 g BW	0.22 ± 0.01^a^	0.16 ± 0.01^c^	0.19 ± 0.01^b^	0.22 ± 0.01^a^	0.001
Heart, g/100 g BW	0.40 ± 0.01	0.37 ± 0.01	0.40 ± 0.01	40 ± 0.01	0.126
Abdominal fat, g/100 g BW	1.14 ± 0.04^b^	1.45 ± 0.09^a^	1.28 ± 0.04^ab^	1.16 ± 0.08^b^	0.012

^a–c^The means values placed at the rows by different superscript letters are significantly different (P < 0.05). Values are expressed as means ± standard error. Abbreviations: (A) control negative (basal diet), (B) control positive (basal diet supplemented with clostridia infection), (C) control positive group diet with 100 g DETOXIZYME/ton of feed, (D) control negative diet group with 100 g DETOXIZYME/ton of feed.

### Blood parameters analysis

The results related to the effect of supplementation of dietary DETOXIZYME under clostridia infection on blood parameters analysis are documented in Table 4. ALT, AST, uric acid, creatinine, and MDA values were decreased (P < 0.05) in chickens fed on basal diets fortified with DETOXIZYME as opposed to the other treatment groups during the age of 35 days. However, birds fed the basal diet fortified with 100 g/ton of DETOXIZYME in the overall period increased calcium, phosphate, zinc, and GPx concentrations compared to other groups.

**Table 4:** Effect of DETOXIZYME supplementation on blood parameters under clostridia infection in Cobb 500 broilers.

Item	Experimental diets				*P*-value
	A	B	C	D	
ALT, U/I	14.8 ± 0.16^b^	17.66 ± 0.21^a^	10 ± 0.11^c^	6.5 ± 0.22^d^	0.001
AST, U/I	56.7 ± 0.71^b^	84.0 ± 1.3^a^	33.7 ± 0.66^c^	14.0 ± 1.8^d^	0.001
Uric acid, mg/dl	1.81 ± 0.005^b^	2.31 ± 0.04^a^	1.43 ± 0.008^c^	1.14 ± 0.03^d^	0.003
Creatinine, mg/dl	0.38 ± 0.01^b^	0.485 ± 0.007^a^	0.30 ± 0.002^c^	0.228 ± 0.008^d^	0.004
Calcium, mg/dl	7.395 ± 0.01^c^	7.075 ± 0.01^d^	7.81 ± 0.014^b^	8.19 ± 0.02^a^	0.001
Phosphate, mg/dl,	6.09 ± 0.01^c^	5.32 ± 0.017^d^	6.62 ± 0.015^b^	7.4 ± 0.015^a^	0.002
Zinc, mg/dl	11.74 ± 0.017^c^	11.03 ± 0.02^d^	12.86 ± 0.015^b^	13.59 ± 0.02^a^	0.001
MDA, nmol/g	2.21 ± 0.02^b^	2.72 ± 0.01^a^	1.61 ± 0.02^c^	1.14 ± 0.01^d^	0.001
GPx, U/mg	406 ± 7^c^	279 ± 2^d^	539 ± 6^b^	653 ± 4^a^	0.001

^a–c^The means values placed at the rows by different superscript letters are significantly different (P < 0.05). Values are expressed as means ± standard error. Abbreviations: (A) control negative (basal diet), (B) control positive (basal diet supplemented with clostridia infection), (C) control positive group diet with 100 g DETOXIZYME/ton of feed, (D) control negative diet group with 100 g DETOXIZYME/ton of feed.

### Plasma biochemical lipids

As shown in Table 5, feed supplementation of DETOXIZYME into Cobb 500 broilers diets under clostridia infection during the trial period reduced (P < 0.05) plasma total cholesterol, and LDL values contrary to the other treatments. While triglyceride was significantly lowered (P < 0.05) by the supplementation of DETOXIZYME in contrast to the control positive group. However, chickens took the basal diet supplemented with 100 g/ton of DETOXIZYME increased HDL compared to other treatment groups in the overall period.

**Table 5:** Effect of DETOXIZYME supplementation on plasma lipids under clostridia infection in Cobb 500 broilers.

Item	Experimental diets				*P*-value
	A	B	C	D	
Total cholesterol, mg/dl	266 ± 11^b^	328 ± 9^a^	232 ± 3^c^	177 ± 9^d^	0.001
Triglyceride, mg/dl	103 ± 1^b^	186 ± 24^a^	94 ± 2^b^	67 ± 2^b^	0.010
LDL, mg/dl	70 ± 4^b^	102 ± 3^a^	45 ± 3^c^	28 ± 3^d^	0.001
HDL, mg/dl	27 ± 1^c^	12 ± 0.5^d^	39 ± 2.2^b^	60 ± 2^a^	0.003

^a–c^The means values placed at the rows by different superscript letters are significantly different (P < 0.05). Values are expressed as means ± standard error. Abbreviations: (A) control negative (basal diet), (B) control positive (basal diet supplemented with clostridia infection), (C) control positive group diet with 100 g DETOXIZYME/ton of feed, (D) control negative diet group with 100 g DETOXIZYME/ton of feed.

### Immunity evaluation

Table 6 documents the results related to the effect of supplementation of DETOXIZYME treatment on immunity. Broilers took the basal diet treated with 100 g/ton of DETOXIZYME increased total protein, albumin, and globulin concentrations in the overall period as opposed to other groups. Although, supplementation of DETOXIZYME at 28 and 35 d of age increased ND antibody titers contrary to other groups. However, there were no significant variances in ND antibody titers were observed due to DETOXIZYME dietary treatments during 7, 14, and 21 days of age.

**Table 6:** Effect of DETOXIZYME supplementation on immunity under clostridia infection in Cobb 500 broilers.

Item	Experimental Diets				*P*-value
	A	B	C	D	
Total protein, mg/dl	2.6 ± 0.02^c^	2.18 ± 0.004^d^	3.055 ± 0.02^b^	3.45 ± 0.02^a^	0.001
Albumin, mg/dl	1.598 ± 0.01^b^	1.461 ± 0.007^c^	1.70 ± 0.013^a^	1.26 ± 0.03^d^	0.001
Globulin, mg/dl	1.505 ± 0.03^b^	0.995 ± 0.01^c^	0.721 ± 0.01^d^	2.19 ± 0.050^a^	0.001
Albumin / Globulin, mg/dl	1.608 ± 0.01^b^	2.03 ± 0.04^a^	1.26 ± 0.004^c^	0.580 ± 0.03^d^	0.001
ND, titer, 7d	3.45 ± 0.4	3.45 ± 0.3	3.62 ± 0.4	3.45 ± 0.4	0.983
ND, titer, 14d	2.67 ± 0.12	2.77 ± 0.21	3.23 ± 0.16	3.05 ± 0.24	0.187
ND, titer, 21d	2.55 ± 0.3	2.4 ± 0.3	2.6 ± 0.2	3.0 ± 0.24	0.362
ND, titer, 28d	2.67 ± 0.19^bc^	2.47 ± 0.25^c^	3.05 ± 0.09^ab^	3.33 ± 0.17^a^	0.016
ND, titer, 35d	2.88 ± 0.19^bc^	2.53 ± 0.20^c^	3.33 ± 0.28^ab^	3.72 ± 0.14^a^	0.005

^a–c^The means values placed at the rows by different superscript letters are significantly different (P < 0.05). Values are expressed as means ± standard error. Abbreviations: (A) control negative (basal diet), (B) control positive (basal diet supplemented with clostridia infection), (C) control positive group diet with 100 g DETOXIZYME/ton of feed, (D) control negative diet group with 100 g DETOXIZYME/ton of feed.

### Bacteriological counts

Table 7 provides information regarding the impact of feed supplementation with DETOXIZYME during clostridium infection on bacteriological counts for broilers. Dietary supplementation of DETOXIZYME into Cobb 500 broilers diets under clostridia infection decreased (P < 0.05) clostridia and *E. coli* bacteria counts in contrast to the control positive treatment group during 35 d of age.

**Table 7:** Effect of DETOXIZYME supplementation on Clostridia and E Coli accounts under clostridia infection in Cobb 500 broilers.

Item	Experimental diets				*P*-value
	A	B	C	D	
Clostridia, log 10 cfu/g	42 × 10^6^ ± 3^b^	58 × 10^6^ ± 2^a^	6 × 10^6^ ± 1^d^	11 × 10^6^ ± 1^c^	0.001
E coli, log 10 cfu/g	13 × 10^6^ ± 0.60^b^	31 × 10^6^ ± 2.9^a^	5.12 × 10^6^ ± 0.5^c^	9.3 × 10^6^ ± 0.66^bc^	0.001

^a–c^The means values placed at the rows by different superscript letters are significantly different (P < 0.05). Values are expressed as means ± standard error. Abbreviations: (A) control negative (basal diet), (B) control positive (basal diet supplemented with clostridia infection), (C) control positive group diet with 100 g DETOXIZYME/ton of feed, (D) control negative diet group with 100 g DETOXIZYME/ton of feed.

### Gut shistopathology

**Figure 1:**
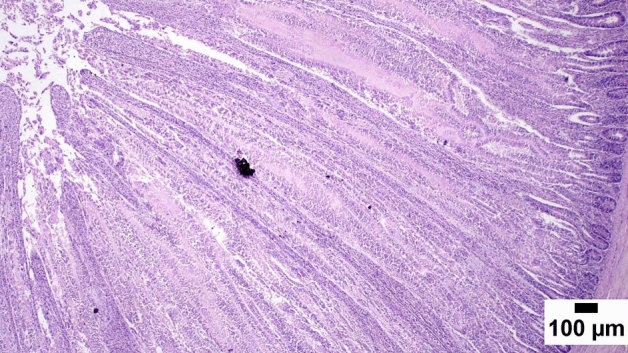
Photomicrograph of the duodenum, A group at 35 days showing normal histology of intestinal mucosa (H&E).

**Figure 2:**
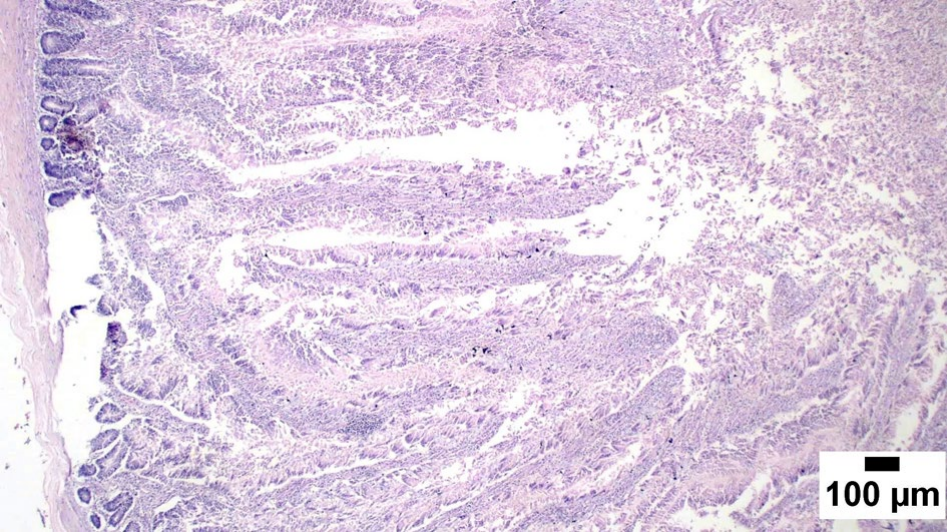
Photomicrograph of the duodenum, B group at 35 days showing marked enteritis with numerous inflammatory cells infiltration in the lamina propria and submucosa.

**Figure 3:**
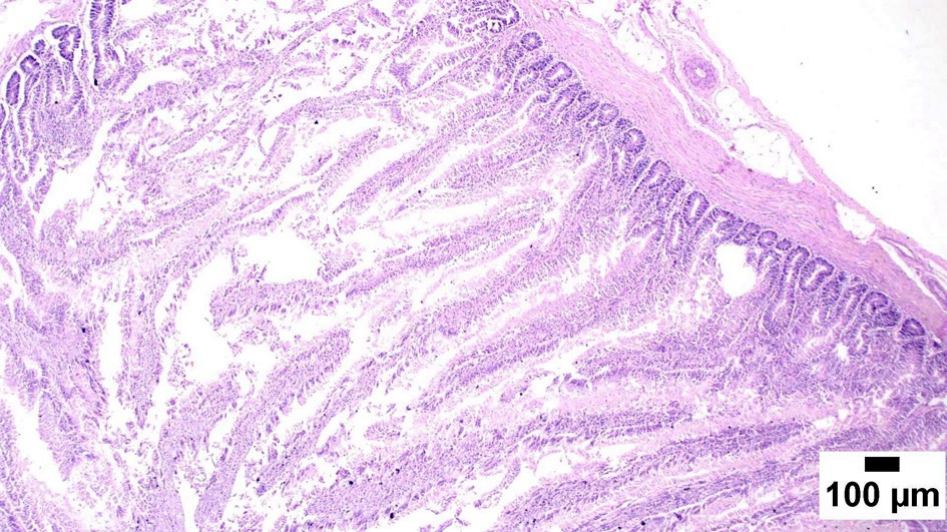
Photomicrograph of the duodenum, B group at 35 days showing atrophied intestinal villi (H&E).

**Figure 4:**
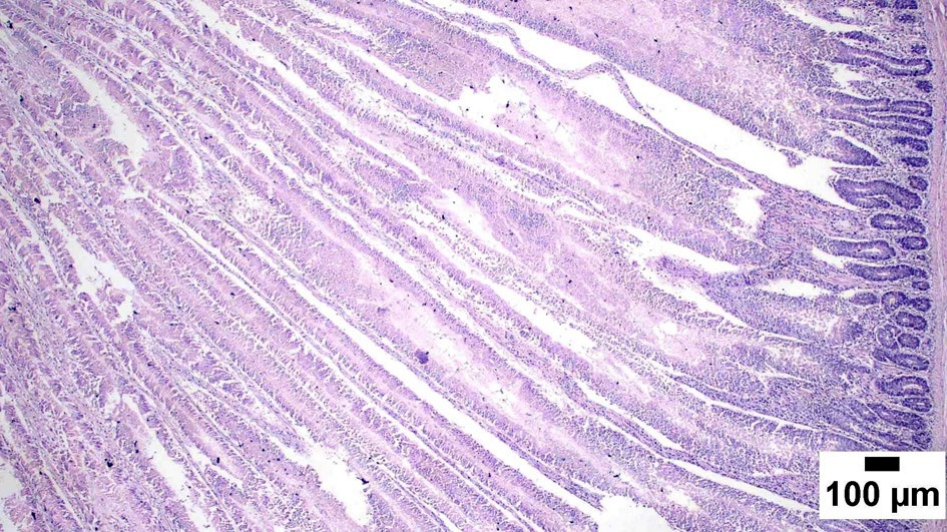
Photomicrograph of the duodenum, C group at 35 days showing ostensibly normal intestinal villi (H&E).

**Figure 5:**
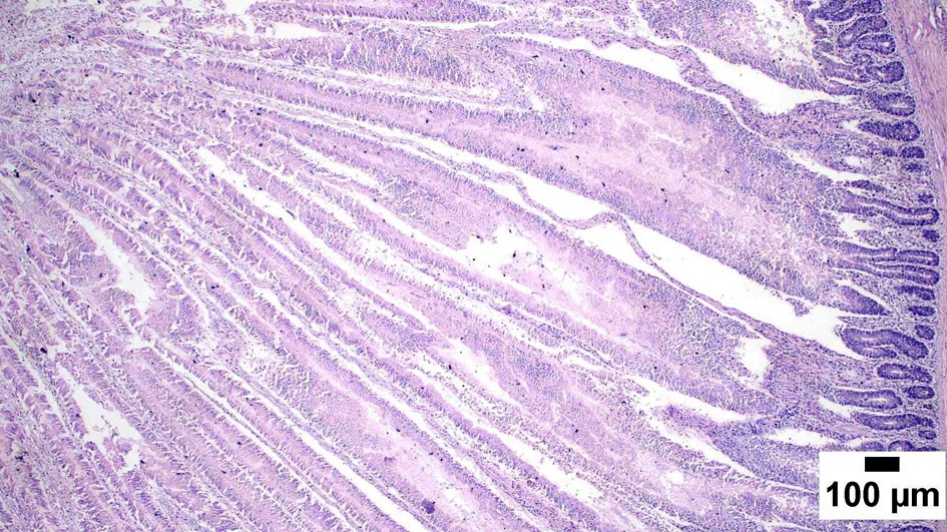
Photomicrograph of the duodenum, C group at 35 days showing long well-oriented intestinal villi with mild inflammation (H&E).

**Figure 6:**
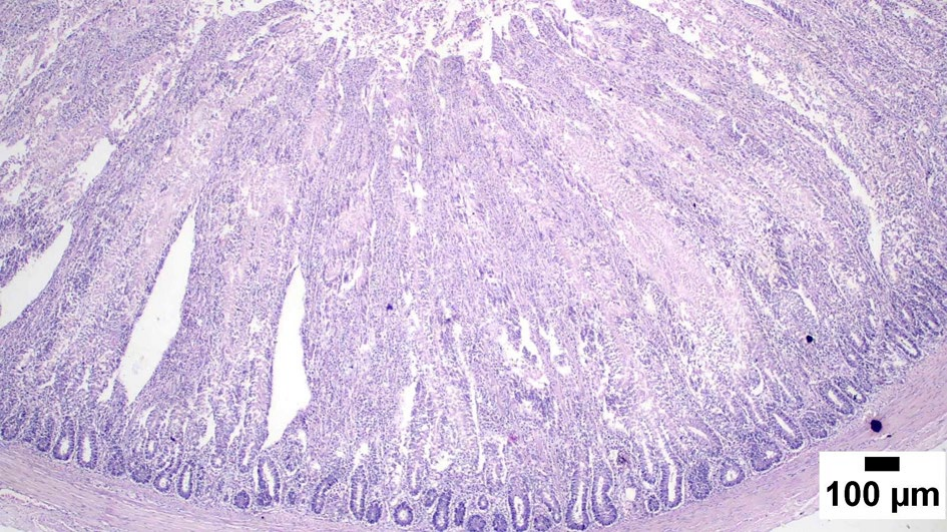
Photomicrograph of the duodenum, D group at 35 days showing ostensibly normal intestinal villi (H&E).

The light microscope examination of the duodenal mucosa showed a normal histological structure of the A and D groups. Severe enteritis was detected in examined sections from group B. Meanwhile, marked improvement is noticed in group C (Figs. 1, 2, 3, 4, 5, 6). Histomorphological examination of duodenal segments showed a significant increase in villi height of group C compared to other groups. Meanwhile, a significant reduction in the V/C ratio was identified in group B in comparison with other groups (Figs. 1, 2, 3, 4, 5, 6).

## Discussion

### Direct effect of anti-toxin multienzymes on birds’ performance

The primary function of the multienzymes is to degrade and detoxify the endo metabolically- and exotoxin content in the broiler's feed, which enhances growth performance^34^. The current findings show that dietary broiler supplementation with 100 g of DETOXIZYME/ton of feed significantly improved BW, BWG, FI, EPEF, and FCR, as well as reduced mortality rate (P < 0.05, Table 2). Regarding the supplementation of multienzymes and their potential impacts on the performance of the production of broilers, inconsistent and contradictory results have been observed. The present outcomes are not concordant with that of Ademola et al.^38^. They reported that the chickens fed a meal enriched with DETOXIZYME did not exhibit any appreciable variations in broiler development performance due to the high dose of maize mycotoxins contamination present before the experiment's start^38^. On the other hand, Schatzmayr et al. declared that broilers’ growth performance could be improved by feed supplementation with bacterial enzymes that can detoxify mycotoxin to a harmless form in the animal's GIT before absorption^30^. In another experiment, Bedford et al. indicated that exogenous enzyme supplementation in animal feed diets speeds up the breakdown of various antinutritional components, which raises the value of the nutrients in the feed, improves growth efficiency, and increases animal feed efficiency^46^. Farmers must improve and maintain optimum digestive health in their birds since it leads to an enhanced performance index, enhanced feed utilization, and improved weight gain^47^. By breaking down macromolecules, modifying broiler gut physiology, and modifying the bacterial composition, multienzyme would enhance digestive health and increase the digestibility of nutrients in broilers^48^. This boost in growth performance may be triggered by the impacts of the reduction in the clostridia count and its secreted endotoxin, as well as the removal of the mycotoxins and their bad effects.

### Indirect effect of anti-toxin multienzymes on carcass and internal organs weight

As shown in Table 3, adding multienzymes to broilers’ diet unprotected from clostridia infection did not disturb the weight of the breast or thigh. However, carcass and spleen were significantly advanced in the multienzymes-treated group (P < 0.05) contrary to the clostridia infection-treated group. This increase in carcass and spleen weight may indicate the beneficial impacts of multienzymes supplementation. Our findings argue with those reported by Mohammed et al. who informed that enzyme addition in chicken diets did not impact carcass and meat quality measures, except for breast meat weight^49^. Furthermore, while a low protein diet led to high carcass weight, the addition of multienzymes to a high ME diet for broilers had no impact on carcass composition, organ weights, or meat quality^50,51^. However, Taheri et al. found that multienzyme supplementation to chicken feed increases nutrient availability and digestibility, which can enhance carcass weight because of increased nutrient utilization^52^. Findings from other studies, however, revealed that diet had no discernible impact on abdominal fat^53^.

### Indirect effect of anti-toxin multienzymes on blood parameters

As shown in Table 4, dietary supplementation with multienzymes significantly decreased the ALT, AST, uric acid, creatinine, and MDA levels in plasma, while calcium, phosphate, zinc, and GPx significantly increased (P < 0.05). It should be noted that aflatoxin hurts some serum enzyme activities such as (GGT, ALP, AST), blood chemistry (globulin, albumin, cholesterol, total proteins), and the weight of the liver. Aflatoxin binders, like aluminum silicate, can lessen the negative effects of aflatoxins by preventing their absorption by attaching toxins inside the bird's gut^54–56^. Previous research by Ademola et al. has shown that combining several enzymes DETOXIZYME in broilers' feed did not significantly change the levels of ALT and AST, although it did cause the concentration of uric acid to drop^38^. According to Attia et al., comparing the multienzyme supplementation group to the control treatment, the levels of ALT, AST, and MDA were significantly decreased^57^. Plasma MDA Reduction is a positive impact of supplementing anti-toxin multienzymes that destruct endogenous toxins and indirectly decrease oxidative stress, increasing GPx levels and, leading to decreased MDA levels. Cowieson et al. and Yang et al. discovered that consuming a multienzyme compound comprising carbohydrases and proteases increased energy utilization, protein, P, and Ca in broiler chicks^36,37^.

### Indirect effect of anti-toxin multienzymes on plasma total lipids

The plasma lipid profile is an important factor in lipid metabolism balance. Based on this, it's crucial to understand that clostridia produce alpha toxins, endotoxins that cause the source of cholesterol to attach to theta-toxin, also identified as perfringolysin^58^. The cholesterol-dependent cytolysin family includes the perfringolysin toxin. Venom family members have comparable biological characteristics and share between 40 and 80% of their structural identity^59^. The thiol-activated cytolysin family of toxins, which include perfringolysin, are also secreted by gram-positive bacteria and can complement the actions of alpha toxins^60^. By inserting a transmembrane domain, the perfringolysin O generated by *C. perfringens* oligomerizes on the cell membrane surface where there is a supply of cholesterol, creating a pore^61^. This allows ions and macromolecules to enter and exit the cell^60^. Furthermore, the Net-B toxin is boosted when there is a source of cholesterol. However, the toxin's receptor on the living cell is yet unclear^59,62^. Similar to the current findings, Attia et al. discovered that broiler diets with added anti-toxin multienzymes reduced plasma cholesterol and LDL while raising HDL and albumin^57^. To the contrary, El-Katcha et al. testified that when compared to chickens who took the same feed without the addition of enzymes, the concentrations of cholesterol and triglycerides were not significantly altered^63^. Indicating that enzymes had a favorable impact on blood cholesterol, the various plasma cholesterol, and HDL to LDL ratios were greater in the control treatments than in the groups that received multienzyme supplementation^64–66^. These beneficial impacts of multienzyme supplementation on plasma lipid metabolites warrant further research.

### Indirect effect of anti-toxin multienzymes on immunity

As shown in Table 6, dietary supplementation of multienzymes DETOXIZYME increased the serum total protein, globulin, and ND antibody titers, and decreased the albumin and albumin/globulin ratio. The effects of multienzymes DETOXIZYME on birds' immunity have been studied before. Previous studies showed that aflatoxicosis is linked to decreased immunological response^24,67^. Other researchers found that consumption of aflatoxin is associated with poor performance brought on by a reduction in immune response, as well as liver cirrhosis linked to alterations in blood biochemistry, the formation of clinical diseases, and increased mortality in broiler chickens^16,17^. In agreement with that, Liu et al. found that incorporating multiple anti-toxin enzymes enhances the immunological response, lowers *C. perfringens* counts, and lowers antibodies against aflatoxins in broilers^33^. Moreover, when added to the feed of pigs exposed to fumonisin, anti-toxin multienzymes lessened the harmful effects of mycotoxins on their liver, lungs, and jejunum while also boosting their immune system^68,69^. Increased nutritional availability due to multiple enzyme supplementations was complemented by better immunological function^37,65,70^.

### Direct effect of anti-toxin multienzymes on gut bacteriological counts

As presented in Table 6, feed supplementation with the multienzyme DETOXIZYME reduced clostridia and *E. coli* bacterial counts. The effects of multienzymes on clostridia and *E. coli* intestinal bacteria have been studied before. Scientists have suggested several methods as potential substitutes for antibiotics in poultry feed to manage the inflammatory clostridia that reduce productivity^71,72^. In this context, the use of anti-toxin multienzymes in broiler feeds is one of these tactics^34^. In chicken feed manufacturing, anti-toxin multienzymes are fortified to contaminated feed in the broiler sector to lessen the harmful effects of pathogenic microorganisms such as clostridia, *E. coli*, and *Salmonella* spp.^35^. Gibson et al. found that via promoting lactic acid bacteria development in the hindgut, the hydrolysis products of enzymes may subtly inhibit the growth of some pathogens^73^. However, the current findings contradict those of Madigan-Stretton et al. and Lourenco et al. who revealed no significant prevalence for any connected bacterial species and no variations in microbial diversity across all multienzyme natuzyme treatment groups^48,74^.

### Direct effect of anti-toxin multienzymes on gut histopathology

As presented in Figs. 1, 2, 3, 4, 5, and 6, dietary supplementation of anti-toxin multienzymes shows an increase in villi height especially in C and D groups compared to other groups. In the same line, Aghili et al. demonstrated that the jejunum's villous height and crypt depth could be considerably improved by supplementing it with a high dose of enzymes (P < 0.05)^75^. Madigan-Stretton et al. discovered that villus height, width, and crypt depth were all improved in the duodenum by super-dosing multienzymes^48^. Villus width and the number of goblet cells in the jejunum were also increased. Furthermore, the supplementation of natuzyme multienzymes to corn-soybean meal significantly improved villi height and enhanced nutrient utilization^76^. Shakouri et al., Ahmed et al., and Mazhari et al. found that multienzymes were added, but they didn't overdose, and they significantly increased crypt depth and villus height^77–79^. However, Teirlynck et al. revealed that incorporating wheat into a diet resulted in villi fusion and mucosal damage, both of which are symptoms of an inflammatory bowel illness^80^. As demonstrated by the extension of the intestinal villi, the enhanced release of nutrients brought on by enzyme supplementation increased the nutrients accessible for absorption, shifting the biochemical outcome in favor of the anabolic reaction and muscular growth^81^.

## Conclusions

Anti-toxin multienzymes blend (DETOXIZYME) supplementation can destroy the bacterial endo and exotoxin in broilers’ gut and reduce the count of clostridia. Moreover, it can improve weight gain, biochemistry of blood, bacterial counts, and gut histomorphology in broiler chickens. As a result, supplementing anti-toxin multienzyme (DETOXIZYME) may be a successful and advantageous growth booster, with a dosage of 100 g/ton in a broiler diet under clostridia infection.

## Data Availability

The datasets used and/or analyzed during the current study are available from the corresponding author upon reasonable request.
